# Liver-Specific PGC-1beta Deficiency Leads to Impaired Mitochondrial Function and Lipogenic Response to Fasting-Refeeding

**DOI:** 10.1371/journal.pone.0052645

**Published:** 2012-12-28

**Authors:** Kari T. Chambers, Zhouji Chen, Peter A. Crawford, Xiaorong Fu, Shawn C. Burgess, Ling Lai, Teresa C. Leone, Daniel P. Kelly, Brian N. Finck

**Affiliations:** 1 Department of Medicine, Washington University School of Medicine, St. Louis, Missouri, United States of America; 2 Advanced Imaging Research Center and Department of Pharmacology, University of Texas Southwestern Medical Center, Dallas, Texas, United States of America; 3 The Sanford-Burnham Medical Research Institute at Lake Nona, Orlando, Florida, United States of America; Boston University School of Medicine, United States of America

## Abstract

PGC-1β plays pleiotropic roles in regulating intermediary metabolism and has been shown to regulate both catabolic and anabolic processes in liver. We sought to evaluate the effects of PGC-1β on liver energy metabolism by generating mice with postnatal, liver-specific deletion of PGC-1β (LS-PGC-1β^−/−^ mice). LS-PGC-1β^−/−^ mice were outwardly normal, but exhibited a significant increase in hepatic triglyceride content at 6 weeks of age. Hepatic steatosis was due, at least in part, to impaired capacity for fatty acid oxidation and marked mitochondrial dysfunction. Mitochondrial DNA content and the expression of genes encoding multiple steps in mitochondrial fatty acid oxidation and oxidative phosphorylation pathways were significantly diminished in LS-PGC-1β^−/−^ mice. Liquid chromatography mass spectrometry-based analyses also revealed that acetylcarnitine and butyrylcarnitine levels were depleted whereas palmitoylcarnitine content was increased in LS-PGC-1β^−/−^ liver, which is consistent with attenuated rates of fatty acid oxidation. Interestingly, loss of PGC-1β also significantly impaired inducible expression of glycolytic and lipogenic enzymes that occurs with high carbohydrate diet refeeding after a prolonged fast. These results suggest that PGC-1β plays dual roles in regulating hepatic fatty acid metabolism by controlling the expression of programs of genes involved in both fatty acid oxidation and de novo fatty acid synthesis.

## Introduction

Transcriptional coactivators are a class of proteins that regulate gene transcription by interacting with DNA-bound transcription factors. Amongst the numerous coactivators, the peroxisome proliferator-activated receptor γ coactivator 1 (PGC-1) coactivators have been strongly linked to the regulation of intermediary metabolism. The first member of the PGC-1 family, PGC-1α, was identified in a screen to identify proteins that interacted with PPARγ and were enriched in brown adipocytes [1]. Based on sequence homology in key domains, a second member of the PGC-1 family, PGC-1β, was subsequently identified [2]. A more distantly related PGC-1 family member (PRC1) has also been cloned, but has been less well studied [3]. Since their original discovery, several nuclear receptor and non-nuclear receptor transcription factors have been identified as targets for coactivation by PGC-1α and β [4], [5], [6].

Gain-of-function and loss-of-function approaches have demonstrated that PGC-1α and PGC-1β play important roles in regulating oxidative metabolism. PGC-1s coactivate a variety of transcription factors including PPARs, estrogen-related receptors, and nuclear respiratory factors to drive expression of genes encoding enzymes involved in fatty acid oxidation and oxidative phosphorylation [4], [5], [6]. Experiments conducted using methods to overexpress PGC-1α or β uniformly demonstrate an increase in the capacity for oxidative metabolism and suggest that these two coactivators activate a generally similar pattern of metabolic gene expression. Mice in which either PGC-1α or PGC-1β have been constitutively deleted are relatively normal at baseline, but respond abnormally to various physiologic and metabolic stimuli [7], [8], [9], [10], [11]. On the other hand, mice with constitutive deletion of both PGC-1α and PGC-1β die soon after birth, due at least in part to cardiac failure [12], indicating that this system is absolutely required for postnatal life. Collectively, these results are consistent with the idea that PGC-1α and β are functionally redundant for basal metabolic homeostasis, but that in the context of stimuli that elicit a robust metabolic response, full activity of the PGC-1 system is required for the appropriate metabolic adaptation.

Whereas PGC-1α and β play mostly overlapping roles in regulating β-oxidation and oxidative phosphorylation, there are other examples of pathways that are specific to one of these proteins. For example, PGC-1α regulates expression of genes involved in gluconeogenesis through coactivation of HNF4α and FOXO1, while PGC-1β can neither coactivate these transcription factors nor regulate the expression of gluconeogenic genes [13], [14]. Conversely, PGC-1β, but not PGC-1α, overexpression induces several genes involved in the process of de novo lipogenesis [15]. This may be explained explained, in part, by PGC-1β-mediated activation of sterol response element binding protein (SREBP1), which is a principal and direct regulator of these genes [16].

Three distinct lines of mice with constitutive knockout of PGC-1β [9], [10], [12] and a fourth mouse line harboring a hypomorphic PGC-1β allele [11] have been generated and characterized. Two of these models, including the PGC-1β hypomorph, were reported to exhibit hepatic steatosis and evidence for diminished mitochondrial oxidative metabolism [10], [11]. Another line exhibited hepatic steatosis only when challenged with physiological or dietary stimuli, such as high fat diet [9]. Thus, the reported severity of the hepatic phenotypes of these mouse lines has varied somewhat, which could be explained by mouse background strain, epigenetic effects, or environmental variation. Many models of constitutive knockout are also complicated by chronic compensatory mechanisms. Furthermore, since PGC-1β is deficient in all tissues, interorgan crosstalk and peripheral organ contribution to the hepatic phenotype may explain some aspects of the observed phenotype.

To address these issues and to further evaluate the direct effects of PGC-1β on hepatic energy metabolism, we generated mice with liver-specific deletion of PGC-1β by using *Cre*-LoxP methodology and characterized the hepatic phenotype of these mice. Mice with liver-specific, postnatal PGC-1β deficiency exhibited hepatic steatosis and marked impairments in mitochondrial oxidative capacity. Interestingly, the present data support dual roles for PGC-1β in regulating hepatic fatty acid homeostasis, since loss of PGC-1β also blunted the increase in lipogenesis that occurs during refeeding after a prolonged fast. These findings demonstrate that PGC-1β is a critical regulator of these pathways in liver.

## Experimental Procedures

### Ethics Statement

All animal experiments were approved by the Animal Studies Committee of Washington University School of Medicine.

### Animal Studies

The generation of mice harboring PGC-1β alleles containing LoxP sites flanking exons 4 through 6 has been described previously [12]. To drive liver-specific knockout of PGC-1β, PGC-1β flox/flox mice were crossed with hemizygous transgenic mice expressing *Cre* recombinase under control of the albumin promoter (Alb-*Cre*) (Figure S1). In all experiments, 6–8 week old mice were utilized and mice transgenic for *Cre* recombinase in liver and harboring two PGC-1β floxed alleles (liver-specific PGC-1β knockout (LS-PGC-1β^−/−^ mice)) were compared to sex-matched littermate PGC-1β flox/flox mice not expressing *Cre* and are therefore essentially wild-type (WT). For tissue and plasma collection, mice were sacrificed by CO_2_ gas asphyxiation at approximately 0900 h, which is 3 h after the initiation of the light phase. For fasting-refeeding studies, individually-housed mice were fasted for 24 h and then either refed with high sucrose (60% calories) for 16 h or continued fasting for another 16 h. Tissues were snap frozen for future analyses, preserved in 10% formalin for histologic sectioning, or mitochondria were isolated from fresh tissue for use in mitochondrial respiration studies immediately thereafter.

Triglyceride secretion rates were determined by using Triton WR-1339 injection to inhibit lipoprotein lipolysis, as previously described [17]. Plasma triglyceride concentrations were determined at 0, 60, and 120 minutes after injection of Triton WR-1339 and rates of secretion were expressed as mg/kg body weight per h.

### Hepatocyte Studies

Primary cultures of mouse hepatocytes were prepared from mice as described [17]. Rates of fatty acid oxidation, glycolysis, and fatty acid synthesis were assessed 2–3 hours after cells were plated and were performed using [9,10-^3^H]-palmitic acid [18], [19], ^3^H-glucose [20], or ^3^H-acetate [21].

### Plasma and tissue lipid quantification

Plasma triglyceride, free fatty acid, and cholesterol concentrations were determined using colorimetric assays using plasma from ad libitum-fed mice. Plasma glucose concentrations were determined in whole blood using the One-Touch Ultra glucometer. Hepatic triglyceride and cholesterol content was determined in liver lysates by using colorimetric assays from Thermo Scientific as previously described [22].

### Histologic analyses

Formalin-fixed tissues were embedded in paraffin, sectioned, and hematoxylin and eosin stained by the Washington University Digestive Diseases Research Core Center. Stained slides were evaluated and imaged by a trained hepatic histopathologist blinded to treatment groups (Dr. E.B. Brunt).

### Plasma alanine aminotransferase (ALT) and aspartate aminotransferase (AST) quantification

Plasma ALT and AST concentrations were measured by using 10 ml of plasma according to the manufacturer's protocols (Teco Diagnostics). Calculations were performed using the 0 timepoint and 5 minute timepoint, as the assay was found to be linear. The results are presented as U/L.

### Mitochondrial Function

Hepatic mitochondria were isolated from CO_2_ euthanized mice by sucrose gradient centrifugation [23]. Briefly, 150 mg liver samples were excised, rinsed in ice-cold Mitochondrial Isolation Medium (MIM, 10 mM Na HEPES, pH 7.2, 300 mM sucrose, 0.2 mM EDTA). Ten mL of ice cold MIM (pH 7.4) containing BSA (Sigma; 1 mg/mL) was subsequently added to the preparation. The samples were homogenized on ice using a Glas-Col dounce homogenizer and centrifuged at 600× g for 10 min at 4°C. The resulting supernatant, which contained mitochondria, was spun at 8,000× g for 15 min at 4°C, the supernatant discarded, the mitochondrial pellet resuspended in 10 mL of ice cold MIM-BSA, and the sample centrifuged again at 8,000× g for 15 min at 4°C. The pellet was briefly washed in ice cold MIM, and re-suspended in 225 ml of ice cold MIM (pH 7.2). Mitochondrial preparations were maintained on ice and used for respiration assays on the same day as the isolation. Protein content was quantified by Bradford assay (Bio-Rad): 0.5 mg of mitochondrial protein was used for each respiration assay.

Respiration assays were performed at 37°C using a water-jacketed Clark electrode (Hansatech Instruments) and conditions described previously [24]. Briefly, 1 mL of respiration buffer (20 mM HEPES, pH 7.1, 125 mM KCl, 3 mM magnesium acetate, 5 mM KH_2_PO_4_, 0.4 mM EGTA, 0.3 mM DTT, 2 mg BSA/mL) was used to supply one of two substrate combinations: (i) 20 mM palmitoyl-L-carnitine plus 5 mM malate or (ii) 5 mM succinate plus 10 mM rotenone. The solubility of oxygen in the respiration buffer at 37°C was 235 nmol O_2_/mL. Following measurement of basal (state 2) respiration, 1 mM ADP was added to isolated mitochondria in respiration buffer, and maximal (state 3) respiration defined. Thereafter, state 4 (ADP-depleted) respiration was mimicked by adding 1 mg/mL oligomycin to inhibit ATP synthase. Uncoupled respiration was measured using 5 mM FCCP (carbonylcyanide-p-trifluoromethoxyphenylhydrazone, Sigma).

### Western blotting

Total cell proteins were collected using RIPA buffer containing protease and phosphatase inhibitors. Western blotting studies were performed using antibodies directed against PGC-1β (gift of A. Kralli), CPT1a [25], MCAD [26], ATP5B (Santa Cruz Biotechnology, Inc.), citrate synthase (gift of John Holloszy), succinate dehydrogenase subunit A (Abcam Inc.), fatty acid synthase (Abcam Inc.), acetyl-coA carboxylase (pan-specific; Cell Signaling), actin (Sigma-Aldrich), or calnexin (Stressgen).

### Quantitative PCR and RT-PCR

Total RNA was isolated using the RNAzol method (Tel-Test). Genomic and mitochondrial DNA was also isolated using RNAzol by collecting the lower phase followed by back extraction with 4 M guanidine thiocyanate, 50 mM sodium citrate, 1 M tris, and an alcohol precipitation.

Real-time PCR was performed using the ABI PRISM 7500 sequence detection system (Applied Biosystems, Foster City, CA) and the SYBR green kit. Arbitrary units of target mRNA were corrected by measuring the levels of 36B4 RNA. Mitochondrial DNA content was determined by SYBR green analysis (Applied Biosystems). Levels of NADH dehydrogenase subunit 1 (mitochondrial DNA) were normalized to the levels of lipoprotein lipase (genomic DNA). The sequence of the oligonucleotides used in qPCR analyses will be provided upon request.

### Acylcarnitine analysis

Twenty to thirty mg liver samples were homogenized in acetonitrile and acylcarnitines were isolated and quantified as previously described [27] with some minor modification. Tissue acylcarnitines were separated by C18 LC and detected with an API 3200 triple quadrupole LC-MS/MS mass spectrometer (Applied Biosystems/Sciex Instruments) in positive ionization MRM mode. Free carnitine was monitored using the 176 to 117 MRM transition. Acylcarnitines were monitored using a precursor of 99 Da. Acylcarnitines were quantified by comparison with internal ^13^C standards (Cambridge Isotope Laboratories, Inc.).

### Statistical Analyses

Statistical comparisons were made using analysis of variance (ANOVA) or *t*-test. All data are presented as means ± SEM, with a statistically significant difference defined as a *P* value<0.05.

## Results

### Generation of mice with liver-specific deletion of PGC-1β

**Figure 1:**
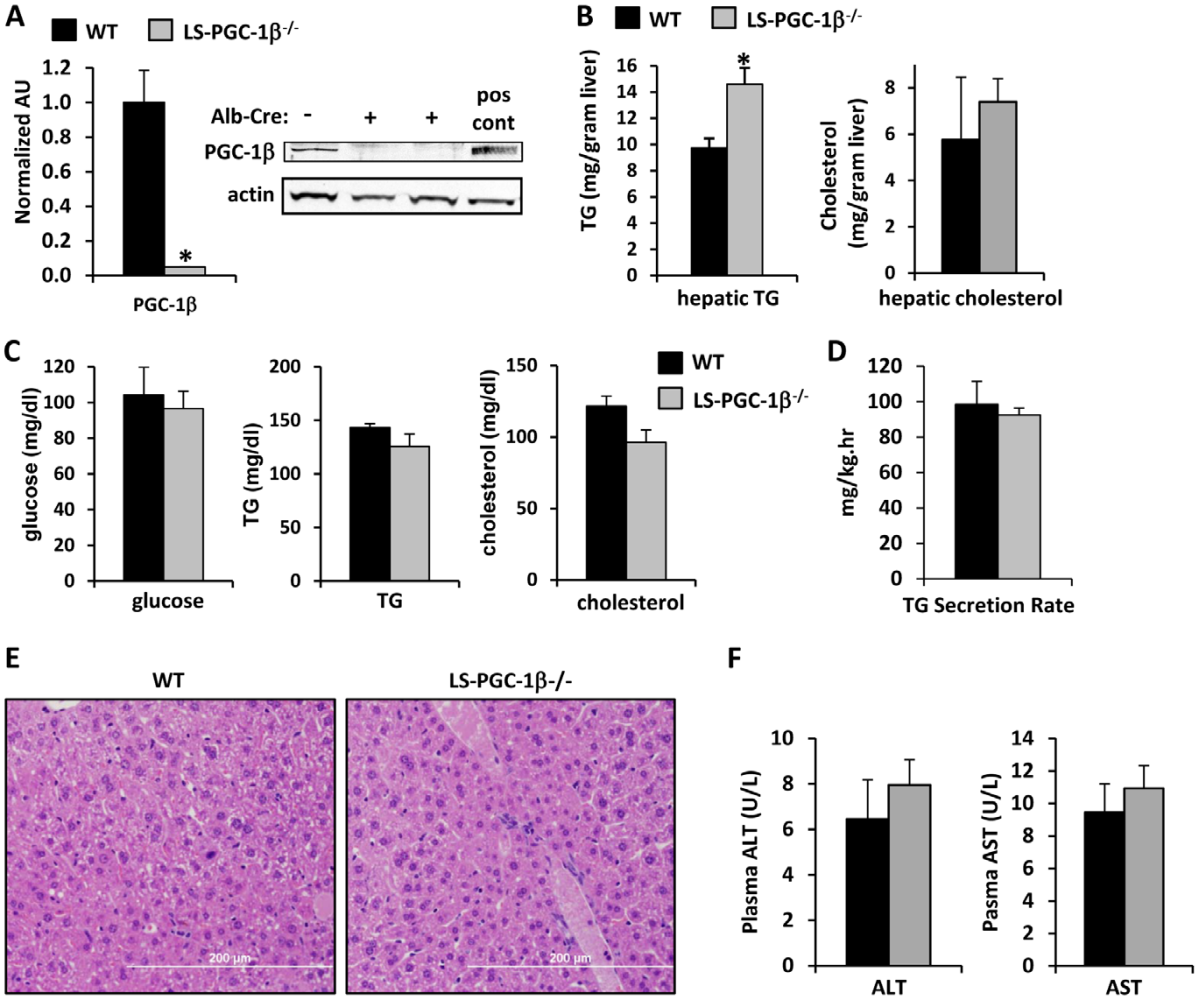
Liver-specific deletion of PGC-1β. [**A**] The graph shows the expression of PGC-1β (Ppargc1b) mRNA in liver of 6 week old female WT (fl/fl) or littermate LS-PGC-1β^−/−^ mice (n = 5). *P<0.05 versus WT mice. Inset is a western blot using liver lysates from the same mice. Lysates from cells infected with an adenovirus to overexpress PGC-1β were used as a positive control. [**B**] The graphs show hepatic triglyceride and cholesterol content in liver of 6 week old WT (fl/fl) or littermate LS-PGC-1β^−/−^ mice (n = 5). [**C**] The graphs represent mean plasma glucose, triglyceride (TG), and cholesterol concentrations in WT and LS-PGC-1β^−/−^ littermate mice. [**D**] The graph depicts rates of TG secretion in WT and LS-PGC-1β^−/−^ littermate mice after injection with Triton WR-1339 to inhibit VLDL lipolysis. [**E**] Representative H&E stained sections of liver from 6 week old WT and LS-PGC-1β^−/−^ littermate mice are shown. [**F**] The graphs represent mean plasma ALT and AST concentrations in WT and LS-PGC-1β^−/−^ littermate mice.

Mice with liver-specific knockout of PGC-1β were generated by crossing mice with loxP sites flanking exons 4–6 (Figure S1) of the PGC-1β allele with transgenic mice expressing *Cre* recombinase in a liver-specific manner (LS-PGC-1β^−/−^ mice). The recombination event generates a coding sequence frameshift, a premature stop codon in original exon 7, and has been shown to lead to complete loss of PGC-1β protein when constitutively knocked out in mice [12]. By 6 weeks of age, PGC-1β flox/flox mice expressing *Cre* in liver exhibited gene recombination (Figure S1) and an almost complete loss of hepatic PGC-1β mRNA and protein (Figure 1A). LS-PGC-1β^−/−^ mice were viable and outwardly normal. However, at sacrifice, we noted that hepatic tissue of LS-PGC-1β^−/−^ mice exhibited a pale appearance indicative of neutral lipid accumulation (data not shown). Indeed, biochemical analyses showed that liver triglyceride levels were significantly increased in LS-PGC-1β^−/−^ mice compared to littermate controls (Figure 1B). No significant differences were observed in hepatic cholesterol content in LS-PGC-1β^−/−^ mice compared to WT littermate controls (Figure 1B). LS-PGC-1β^−/−^ mice did not exhibit any differences in circulating glucose, TG, or cholesterol concentrations (Figure 1C). Rates of triglyceride secretion were also unaltered in LS-PGC-1β^−/−^ mice compared to littermate control mice (Figure 1D). Hematoxylin and eosin staining of livers revealed increased numbers of very small lipid droplets in the livers of LS-PGC-1β^−/−^ mice compared to WT littermate control mice (Figure 1E). However, there was no evidence of increased numbers of infiltrating macrophages or lymphocytes in LS-PGC-1β^−/−^ livers nor was there evidence of fibrotic remodeling, suggesting that the accumulation of triglyceride in LS-PGC-1β^−/−^ mice was not promoting inflammation or liver damage (Figure 1E). Consistent with this, plasma alanine aminotransferase (ALT) and aspartate aminotransferase (AST) concentrations were not significantly different between WT and LS-PGC-1β^−/−^ mice (Figure 1F). These data suggest that postnatal, liver-specific loss of PGC-1β in mice leads to moderate hepatic steatosis, but does not perturb circulating lipid and glucose concentrations or lead to pathological remodeling of the liver.

### Diminished expression of genes involved in mitochondrial oxidative metabolism in LS-PGC-1β^−/−^ liver

**Figure 2:**
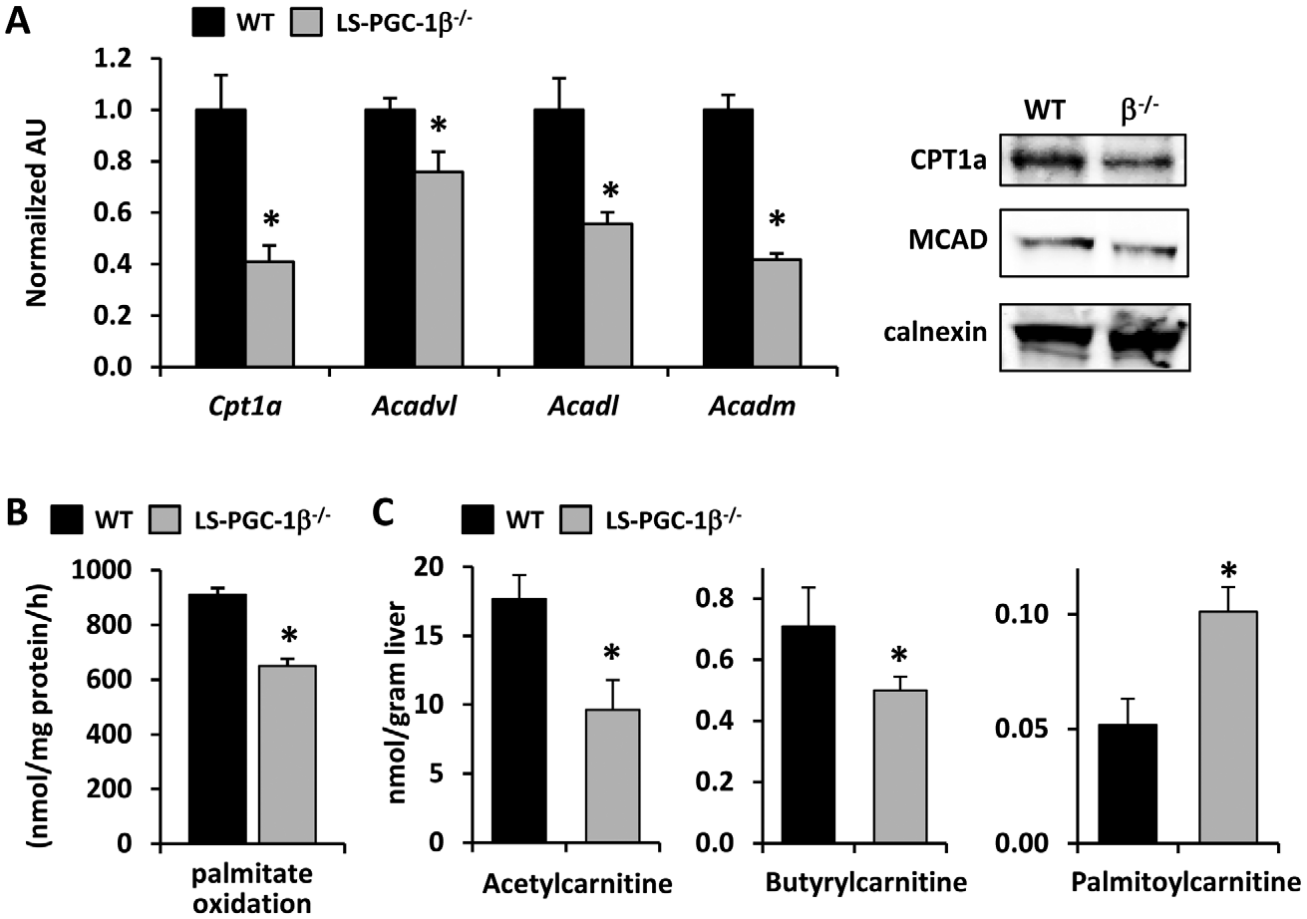
LS-PGC-1β^−/−^ mice have decreased FAO gene expression and decreased rates of palmitate oxidation. [**A**] The graph shows the expression of PGC-1 target gene mRNA involved in FAO in the liver of 6 week old WT (fl/fl) or littermate LS-PGC-1β^−/−^ mice (n = 5). *P<0.05 vs. WT mice. Representative Western blotting analysis of enzymes involved in fatty acid oxidation. [**B**] Rates of palmitate oxidation in hepatocytes isolated from LS-PGC-1β^−/−^ or littermate control mice. *P<0.05 vs. WT mice. [**C**] The graph represents LCMS-based quantification of acyl-carnitine species in the liver of 6 week old female WT (fl/fl) or littermate LS-PGC-1β^−/−^ mice. *P<0.05 vs. WT mice.

Defects in mitochondrial fatty acid oxidation or oxidative metabolism are known to lead to hepatic steatosis [28] and many of these genes are known targets of PGC-1β. We found that the expression of multiple enzymes involved in mitochondrial β-oxidation (*Cpt1a, Acadvl, Acadl, Acadm*) was markedly diminished in LS-PGC-1β^−/−^ mice (Figure 2A). Predictably, rates of fatty acid oxidation were also significantly reduced in hepatocytes from LS-PGC-1β^−/−^ mice (Figure 2B).

We also sought to quantify acylcarnitine levels in hepatic tissues to determine whether altered levels of these intermediates in fatty acid oxidative pathways might further support the conclusion that hepatic fatty acid oxidation was impaired in LS-PGC-1β^−/−^ mice. Liquid chromatography mass spectrometry (LCMS) analyses demonstrated that hepatic content of acetylcarnitine (2 carbon; C2), a product of fatty acid β-oxidation, was diminished in LS-PGC-1β^−/−^ mice compared to WT controls. Butyrylcarnitine (C4) levels were also diminished (Figure 2C), but proprionylcarnitine (C3) and isovalerylcarnitine (C6) were unaffected (data not shown). In contrast, palmitoylcarnitine (C16), a long-chain acylcarnitine, was increased in liver of LS-PGC-1β^−/−^ mice (Figure 2C). This metabolic profile of short-chain acylcarnitine depletion and long-chain acylcarnitine accumulation is consistent with defects in the capacity for fatty acid β-oxidation [29], [30].

**Figure 3:**
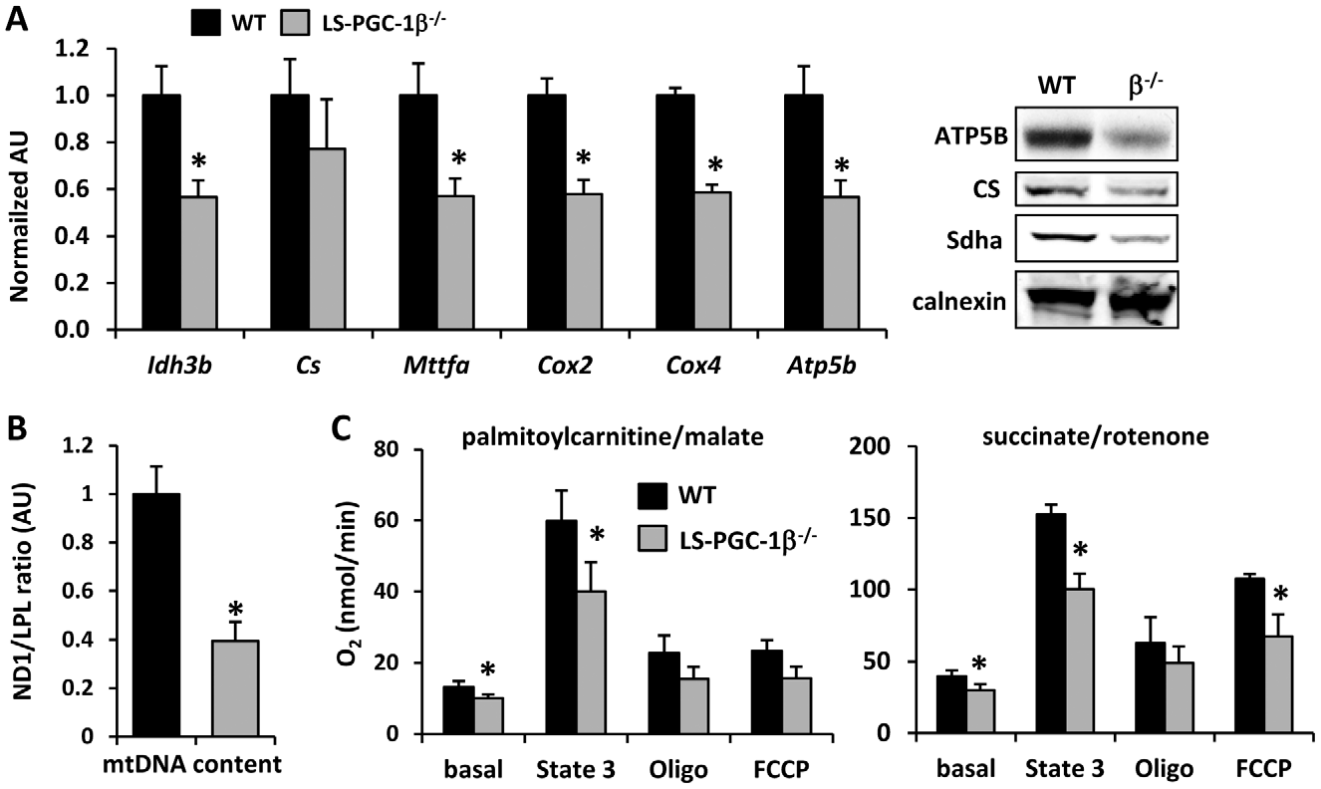
LS-PGC-1β^−/−^ mice exhibit decreased expression of TCA and ETC enzymes and mtDNA content. [**A**] The graph shows the expression of PGC-1 target genes involved in OXPHOS and TCA cycle in the liver of 6 week old female WT (fl/fl) or littermate LS-PGC-1β^−/−^ mice (n = 5). *P<0.05 vs. WT mice. Representative Western blot analysis using antibodies listed at left. [**B**] Mean hepatic mtDNA levels determined by real time PCR analysis shown as arbitrary units (AU; n = 6/group). *P<0.05 vs. WT mice. [**C**] Mitochondrial respiration was performed on isolated mitochondria from the livers of 6 week old WT (fl/fl) or littermate LS-PGC-1β^−/−^ mice (n = 6) and substrates noted.*P<0.05 versus WT mice.

Loss of PGC-1β also led to a significant down-regulation of the expression of the genes encoding the TCA cycle enzyme (*Idh3b*) and several enzymes involved in electron transport chain activity (*Cox2, Cox4, Atp5b*) (Figure 3A). Western blotting studies confirmed that ATP synthase 5B, citrate synthase, and succinate dehydrogenase subunit A (Sdha) protein content were also diminished (Figure 3A). We also observed diminished expression of the transcription factor of activated mitochondrial (*Mttfa*), which controls mitochondrial gene expression and DNA replication (Figure 3A). Consistent with this, the mtDNA to nuclear DNA ratio in LS-PGC-1β^−/−^ liver was significantly diminished compared to control liver (Figure 3B).

Given these data, we evaluated mitochondrial function by quantifying oxygen consumption rates of mitochondria isolated from WT and LS-PGC-1β^−/−^ mice using palmitoylcarnitine or succinate as substrates. These substrates enter oxidative pathways through the fatty acid oxidation and electron transport chain pathways, respectively. Further, the use of palmitoylcarnitine also captures the activity of the TCA cycle. Oxygen consumption rates were markedly depressed compared to WT littermate control mitochondria (Figure 3C). This was most apparent under ADP-stimulated conditions (maximal respiration rates) and was observed using both substrates. Since respiration rates were normalized to mitochondrial protein content, these data suggest that the oxidative capacity per mitochondrion is diminished and are consistent with general hepatic mitochondrial dysfunction in LS-PGC-1β^−/−^ mice.

### Defects in the hepatic response to fasting/refeeding in LS-PGC-1β^−/−^ mice

**Figure 4:**
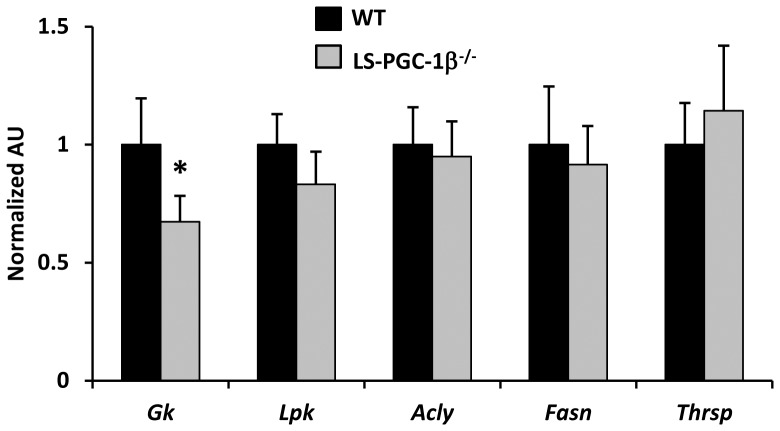
Basal expression of glycolytic and lipogenic genes is normal in LS-PGC-1β^−/−^ mice.

**Figure 5:**
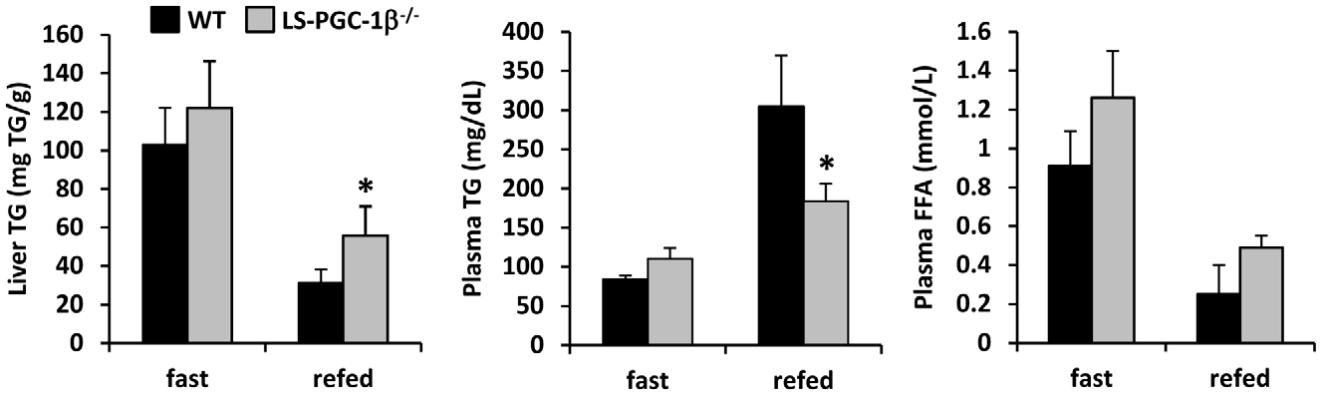
Increased hepatic and circulating TG content in LS-PGC-1β^−/−^ mice in response to fasting-refeeding. The graphs depict hepatic TG content, circulating TG concentration, and plasma free fatty acid concentration in WT and LS-PGC-1β^−/−^ mice after 40 h food deprivation or 24 h fasting and then refeeding for 16 h with high sucrose diet. *P<0.05 versus WT mice.

Previous work has suggested that PGC-1β is involved in the transcriptional regulation of de novo lipogenesis [15]. In six week old mice, the loss of PGC-1β in the liver did not significantly affect the expression of any of the lipogenic genes at baseline, except for moderately-reduced expression of glucokinase (Figure 4). We therefore evaluated the hepatic response to fasting refeeding, which is known to robustly stimulate the expression of many genes involved in glucose uptake and conversion to fatty acids. Littermate WT and LS-PGC-1β^−/−^ mice were fasted 24 h and then either refed with high sucrose (60% total calories) for 16 h or allowed to remain fasting for 16 h more. Forty hours of food deprivation led to a marked increase in hepatic TG content in both WT and LS-PGC-1β^−/−^ mice and levels were not different between genotypes (Figure 5). However, during refeeding, liver TG was increased in the LS-PGC-1β^−/−^ mice compared to WT refed mice. Circulating TG concentration was lower in refed LS-PGC-1β^−/−^ mice compared to WT refed mice, and plasma free fatty acid levels were not significantly different (Figure 5).

**Figure 6:**
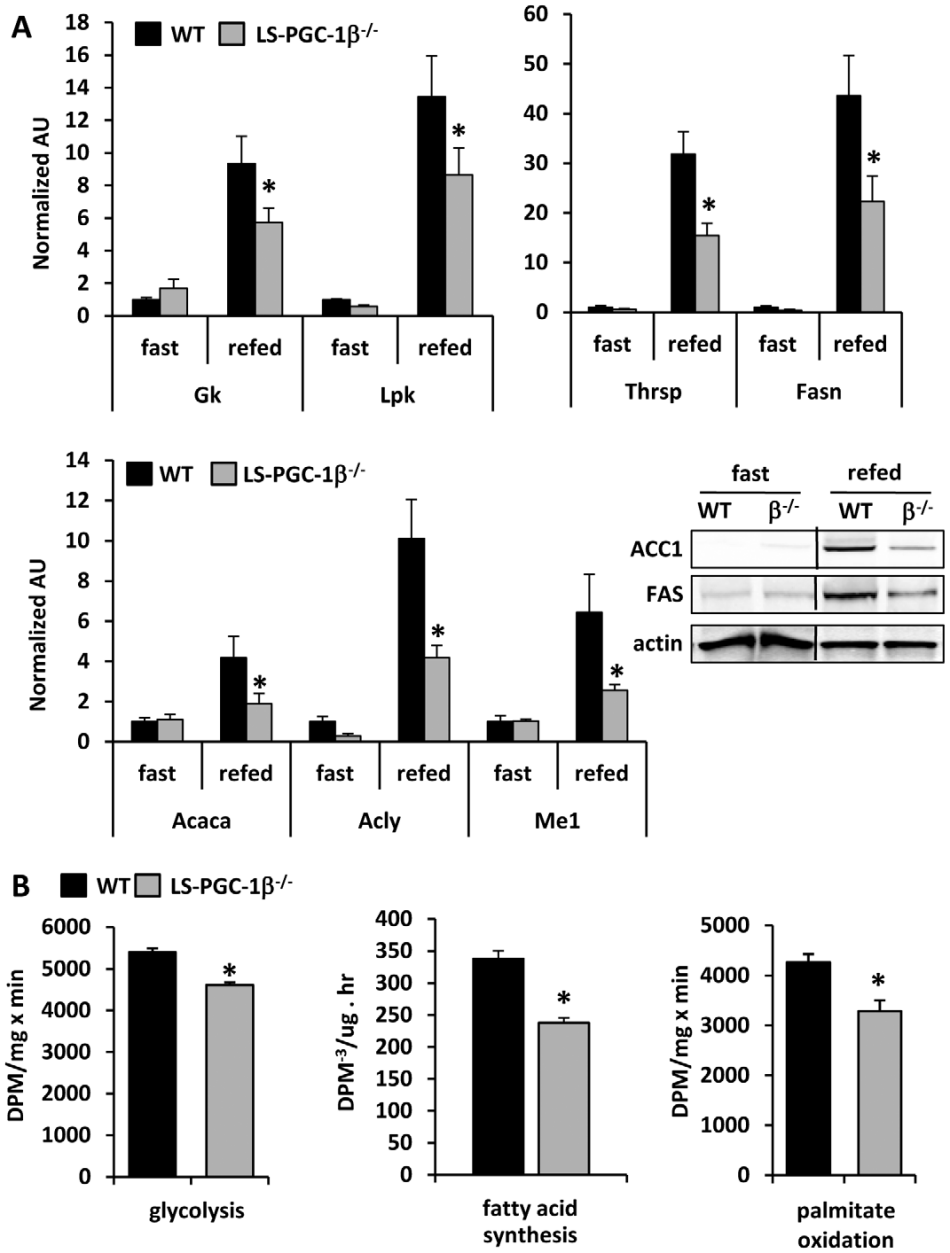
Blunted induction of lipogenic gene expression and lipogenesis in LS-PGC-1β^−/−^ mice in response to fasting-refeeding. The graphs depict the expression of glycolytic and lipogenic gene expression in WT and LS-PGC-1β^−/−^ mice given either fasted 40 h or fasted 24 h and then refed for 16 h with high sucrose diet (n = 6). Representative western blots for corresponding proteins in same mice are shown. *P<0.05 versus WT mice of the same treatment group. The graphs depict the rates of glycolysis, fatty acid synthesis, and palmitate oxidation in hepatocytes from WT and LS-PGC-1β^−/−^ mice after 24 h fasting and then refeeding for 16 h with high sucrose diet. *P<0.05 versus WT mice.

As expected, refeeding caused a marked induction in the mRNA and protein of several enzymes involved in glycolysis (*Gk* and *Lpk*) and lipogenesis (*Thrsp, Fasn, Acca*, *Acly*, and *Me1*) (Figure 6A). However, the response of LS-PGC-1β^−/−^ mice was significantly blunted with regards to the expression of these genes and proteins. Specifically, the induction in the expression of these genes in response to refeeding was reduced by nearly 50% in LS-PGC-1β^−/−^ mice and this finding was confirmed at the level of protein for ACC and FAS, which catalyze the first committed steps in fatty acid synthesis. Given these gene expression results, we also characterized rates of glycolysis and lipogenesis in hepatocytes isolated from mice refed for 16 h. Rates of glycolysis, fatty acid synthesis, and palmitate oxidation were significantly diminished in LS-PGC-1β^−/−^ mice versus WT controls (Figure 6B). Collectively, these data are consistent with PGC-1β playing dual roles in regulating the capacity for fatty acid synthesis and oxidation in liver.

## Discussion

Hepatic energy metabolism is highly regulated at the level of gene transcription. Previous work has suggested that the PGC-1 coactivators play important roles in transcriptionally regulating mitochondrial biogenesis and metabolism in liver. Herein, we evaluated the effects of liver-specific, postnatal PGC-1β knockout on intermediary fatty acid metabolism and mitochondrial oxidative function. The data presented are consistent with dual roles for PGC-1β in regulating intermediary fat metabolism and suggest that PGC-1β controls hepatic mitochondrial biogenesis and oxidative function as well as the capacity for lipogenesis under conditions of high lipogenic flux.

Hepatic steatosis is related to an imbalance between fatty acid influx, de novo synthesis, oxidation, and lipoprotein secretion by the liver. The present data suggest that the steatosis observed in livers of LS-PGC-1β^−/−^ mice is due to impaired hepatic fatty acid oxidation. Six week old LS-PGC-1β^−/−^ mice exhibited marked deficiencies in the expression of genes encoding mitochondrial fatty acid oxidation and electron transport chain enzymes, reduced mtDNA content, and diminished rates of fatty acid oxidation and mitochondrial respiration. Also consistent with a defect in fatty acid β-oxidation, we detected reduced concentrations of short-chain acyl-carnitine while long-chain acyl-carnitine levels were increased. Because this is a liver-specific knockout of PGC-1β, we have eliminated the potentially confounding influences that PGC-1β deficiency in peripheral tissues, such as perturbations in adipose tissue metabolism, might have on hepatic lipid accumulation [31], [32], [33]. The finding that the expression of multiple fatty acid oxidative enzymes is downregulated in LS-PGC-1β^−/−^ mice differs from the phenotype of generalized and liver-specific PGC-1α-deficient mice that exhibit normal expression of these fatty acid oxidative enzymes [8], [34], [35]. While deficits in β-oxidation flux in PGC-1α deficient mice have been observed, these defects are attributed to abnormalities in downstream enzymes involved in electron transport leading to a metabolic bottleneck [35]. The finding that loss of PGC-1β strongly affected β-oxidation enzyme expression may suggest that these genes are more highly influenced by this PGC-1 family member. Studies with isolated mitochondria determined that oxidation of succinate, which enters the mitochondrial electron transport chain directly and downstream of β-oxidation, was also reduced in LS-PGC-1β^−/−^ mice. These data suggest that PGC-1β has broad effects on multiple mitochondrial pathways to coordinate mitochondrial oxidative metabolism.

While previous work has suggested a great deal of overlap between PGC-1α and PGC-1β in the regulation of mitochondrial metabolism, the ability to regulate hepatic de novo lipogenesis has been shown to be a unique feature of PGC-1β. This is likely due to lack of sequence homology between PGC-1β and PGC-1α proteins in the region that mediates the interaction with SREBP1 [15]. In this work, we found that the expression of genes encoding lipogenic enzymes in 6–8 week old LS-PGC-1β^−/−^ mice is normal at baseline, when rates of hepatic de novo lipogenesis would be predicted to be low. However, in the context of refeeding a high carbohydrate diet after a prolonged fast, which is a robust stimulus for lipogenic flux, the inducible expression of genes encoding lipogenic enzymes and rates of lipogenesis were significantly attenuated by loss of PGC-1β. The actions of PGC-1β have also been suggested to be relevant to the lipogenic activation that occurs after feeding a saturated fat-enriched or high fructrose diet [15], [36]. These data suggest that PGC-1β is a regulator of the increased capacity for lipogenesis that occurs in times of nutrient excess, but may not be important for the basal expression of these genes. However, the expression of these lipogenic enzymes was still induced significantly in LS-PGC-1β^−/−^ mice after refeeding, indicating that other transcription factors and/or coactivators are sufficient to mediate a large component of this response. Furthermore, liver TG content was increased, rather than decreased, in refed LS-PGC-1β^−/−^ mice, suggesting that hepatic TG levels in this context are influenced by the capacity to oxidize fatty acids, which is reduced in the LS-PGC-1β^−/−^ mice.

The present data suggest that PGC-1β plays dual roles in governing hepatic fatty acid metabolism and regulates both fatty acid oxidation and de novo fatty acid synthesis [15], [36], which is one of the more puzzling aspects of PGC-1β biology. Why would a factor promote both fatty acid synthesis and degradation, which is in essence, a futile cycle? The process of de novo lipogenesis does require the generation of reducing equivalents that could be produced by fat oxidation. Several known targets of the lipogenic transcription factor, SREBP1, are enzymes that generate reducing equivalents required to drive lipogenesis [16]. High rates of hepatic lipogenesis occur only when nutrients and insulin are in abundance and the organism can afford to spend energy to store fat. There are nutrient-replete physiologic contexts wherein both fatty acid oxidation and fatty acid synthesis would be high, including after administration of high fat diet, which is known to activate PGC-1β [15]. Perhaps with time, clarity regarding the physiological reason for these dichotomous effects will be achieved.

## Supporting Information

Figure S1 **Liver-specific deletion of PGC-1β.** [**A**] The targeting construct and strategy for conditional ablation of PGC-1β is schematized. [**B**] The gel at right shows the results of PCR analyses using the P1 and P3 primers in the schematic at left and DNA isolated from liver of 6 week old PGC-1β fl/fl mice that were expressing Cre recombinase under the control of the liver specific albumin promoter or were non-transgenic for Cre recombinase. Cardiac DNA from one of the transgenic mice is shown as a control.
(TIFF)

## References

[1] Puigserver P, Wu Z, Park CW, Graves R, Wright M, et al (1998) A cold-inducible coactivator of nuclear receptors linked to adaptive thermogenesis. Cell 92: 829–839. 9529258 10.1016/s0092-8674(00)81410-5

[2] Lin J, Puigserver P, Donovan J, Tarr P, Spiegelman BM (2002) Peroxisome proliferator-activated receptor gamma coactivator 1beta (PGC-1beta), a novel PGC-1-related transcription coactivator associated with host cell factor. J Biol Chem 277: 1645–1648. 11733490 10.1074/jbc.C100631200

[3] Andersson U, Scarpulla RC (2001) Pgc-1-related coactivator, a novel, serum-inducible coactivator of nuclear respiratory factor 1-dependent transcription in mammalian cells. Mol Cell Biol 21: 3738–3749. 11340167 10.1128/MCB.21.11.3738-3749.2001 PMC87014

[4] Finck BN, Kelly DP (2006) PGC-1 coactivators: inducible regulators of energy metabolism in health and disease. J Clin Invest 116: 615–622. 16511594 10.1172/JCI27794 PMC1386111

[5] Puigserver P, Spiegelman BM (2003) Peroxisome proliferator-activated receptor-gamma coactivator 1 alpha (PGC-1 alpha): transcriptional coactivator and metabolic regulator. Endocr Rev 24: 78–90. 12588810 10.1210/er.2002-0012

[6] Lin J, Handschin C, Spiegelman BM (2005) Metabolic control through the PGC-1 family of transcription coactivators. Cell Metab 1: 361–370. 16054085 10.1016/j.cmet.2005.05.004

[7] Leone TC, Lehman JJ, Finck BN, Schaeffer PJ, Wende AR, et al (2005) PGC-1alpha deficiency causes multi-system energy metabolic derangements: muscle dysfunction, abnormal weight control and hepatic steatosis. PLoS Biol 3: e101. 15760270 10.1371/journal.pbio.0030101 PMC1064854

[8] Lin J, Wu PH, Tarr PT, Lindenberg KS, St-Pierre J, et al (2004) Defects in adaptive energy metabolism with CNS-linked hyperactivity in PGC-1alpha null mice. Cell 119: 121–135. 15454086 10.1016/j.cell.2004.09.013

[9] Lelliott CJ, Medina-Gomez G, Petrovic N, Kis A, Feldmann HM, et al (2006) Ablation of PGC-1beta results in defective mitochondrial activity, thermogenesis, hepatic function, and cardiac performance. PLoS Biol 4: e369. 17090215 10.1371/journal.pbio.0040369 PMC1634886

[10] Sonoda J, Mehl IR, Chong LW, Nofsinger RR, Evans RM (2007) PGC-1beta controls mitochondrial metabolism to modulate circadian activity, adaptive thermogenesis, and hepatic steatosis. Proc Natl Acad Sci U S A 104: 5223–5228. 17360356 10.1073/pnas.0611623104 PMC1829290

[11] Vianna CR, Huntgeburth M, Coppari R, Choi CS, Lin J, et al (2006) Hypomorphic mutation of PGC-1beta causes mitochondrial dysfunction and liver insulin resistance. Cell Metab 4: 453–464. 17141629 10.1016/j.cmet.2006.11.00 PMC1764615

[12] Lai L, Leone TC, Zechner C, Schaeffer PJ, Kelly SM, et al (2008) Transcriptional coactivators PGC-1alpha and PGC-lbeta control overlapping programs required for perinatal maturation of the heart. Genes Dev 22: 1948–1961. 18628400 10.1101/gad.1661708 PMC2492740

[13] Herzig S, Long F, Jhala US, Hedrick S, Quinn R, et al (2001) CREB regulates hepatic gluconeogenesis through the coactivator PGC-1. Nature 413: 179–183. 11557984 10.1038/35093131

[14] Yoon JC, Puigserver P, Chen G, Donovan J, Wu Z, et al (2001) Control of hepatic gluconeogenesis through the transcriptional coactivator PGC-1. Nature 413: 131–138. 11557972 10.1038/35093050

[15] Lin J, Yang R, Tarr PT, Wu PH, Handschin C, et al (2005) Hyperlipidemic effects of dietary saturated fats mediated through PGC-1beta coactivation of SREBP. Cell 120: 261–273. 15680331 10.1016/j.cell.2004.11.043

[16] Horton JD, Shah NA, Warrington JA, Anderson NN, Park SW, et al (2003) Combined analysis of oligonucleotide microarray data from transgenic and knockout mice identifies direct SREBP target genes. Proc Natl Acad Sci U S A 100: 12027–12032. 14512514 10.1073/pnas.1534923100 PMC218707

[17] Chen Z, Fitzgerald RL, Averna MR, Schonfeld G (2000) A targeted apolipoprotein B-38.9-producing mutation causes fatty livers in mice due to the reduced ability of apolipoprotein B-38.9 to transport triglycerides. J Biol Chem 275: 32807–32815. 10893242 10.1074/jbc.M004913200

[18] Djouadi F, Bonnefont JP, Munnich A, Bastin J (2003) Characterization of fatty acid oxidation in human muscle mitochondria and myoblasts. Mol Genet Metab 78: 112–118. 12618083 10.1016/s1096-7192(03)00017-9

[19] Finck BN, Gropler MC, Chen Z, Leone TC, Croce MA, et al (2006) Lipin 1 is an inducible amplifier of the hepatic PGC-1alpha/PPARalpha regulatory pathway. Cell Metab 4: 199–210. 16950137 10.1016/j.cmet.2006.08.005

[20] Allard MF, Schonekess BO, Henning SL, English DR, Lopaschuk GD (1994) Contribution of oxidative metabolism and glycolysis to ATP production in hypertrophied hearts. Am J Physiol 267: H742–750. 8067430 10.1152/ajpheart.1994.267.2.H742

[21] Lin X, Schonfeld G, Yue P, Chen Z (2002) Hepatic fatty acid synthesis is suppressed in mice with fatty livers due to targeted apolipoprotein B38.9 mutation. Arterioscler Thromb Vasc Biol 22: 476–482. 11884293 10.1161/hq0302.105271

[22] Chen Z, Gropler MC, Norris J, Lawrence JC Jr, Harris TE, et al (2008) Alterations in hepatic metabolism in fld mice reveal a role for lipin 1 in regulating VLDL-triacylglyceride secretion. Arterioscler Thromb Vasc Biol 28: 1738–1744. 18669885 10.1161/ATVBAHA.108.171538 PMC2655237

[23] Boehm EA, Jones BE, Radda GK, Veech RL, Clarke K (2001) Increased uncoupling proteins and decreased efficiency in palmitate-perfused hyperthyroid rat heart. Am J Physiol Heart Circ Physiol 280: H977–983. 11179038 10.1152/ajpheart.2001.280.3.H977

[24] Lehman JJ, Boudina S, Banke NH, Sambandam N, Han X, et al (2008) The transcriptional coactivator PGC-1alpha is essential for maximal and efficient cardiac mitochondrial fatty acid oxidation and lipid homeostasis. Am J Physiol Heart Circ Physiol 295: H185–196. 18487436 10.1152/ajpheart.00081.2008 PMC2494758

[25] Esser V, Kuwajima M, Britton CH, Krishnan K, Foster DW, et al (1993) Inhibitors of mitochondrial carnitine palmitoyltransferase I limit the action of proteases on the enzyme. Isolation and partial amino acid analysis of a truncated form of the rat liver isozyme. Journal of Biological Chemistry 268: 5810–5816. 8449947

[26] Vega RB, Kelly DP (1997) A role for estrogen-related receptor alpha in the control of mitochondrial fatty acid beta-oxidation during brown adipocyte differentiation. J Biol Chem 272: 31693–31699. 9395511 10.1074/jbc.272.50.31693

[27] Millington DS, Kodo N, Norwood DL, Roe CR (1990) Tandem mass spectrometry: a new method for acylcarnitine profiling with potential for neonatal screening for inborn errors of metabolism. J Inherit Metab Dis 13: 321–324. 2122093 10.1007/BF01799385

[28] Leone TC, Weinheimer CJ, Kelly DP (1999) A critical role for the peroxisome proliferator-activated receptor alpha (PPARalpha) in the cellular fasting response: the PPARalpha-null mouse as a model of fatty acid oxidation disorders. Proc Natl Acad Sci U S A 96: 7473–7478. 10377439 10.1073/pnas.96.13.7473 PMC22110

[29] Makowski L, Noland RC, Koves TR, Xing W, Ilkayeva OR, et al (2009) Metabolic profiling of PPARalpha-/- mice reveals defects in carnitine and amino acid homeostasis that are partially reversed by oral carnitine supplementation. FASEB J 23: 586–604. 18945875 10.1096/fj.08-119420 PMC2630792

[30] Sunny N, Satapati S, Fu X, He T, Medibeigi R, et al (2010) Progressive Adaptation of Hepatic Ketogenesis in Mice Fed a High Fat Diet. Am J Physiol Endocrinol Metab 298: E1226–1235. 20233938 10.1152/ajpendo.00033.2010 PMC2886525

[31] Kumar A, Lawrence JC Jr, Jung DY, Ko HJ, Keller SR, et al (2010) Fat cell-specific ablation of rictor in mice impairs insulin-regulated fat cell and whole body glucose and lipid metabolism. Diabetes 59: 1397–1406. 20332342 10.2337/db09-1061 PMC2874700

[32] Wueest S, Rapold RA, Schumann DM, Rytka JM, Schildknecht A, et al (2010) Deletion of Fas in adipocytes relieves adipose tissue inflammation and hepatic manifestations of obesity in mice. J Clin Invest 120: 191–202. 19955656 10.1172/JCI38388 PMC2798678

[33] He W, Barak Y, Hevener A, Olson P, Liao D, et al (2003) Adipose-specific peroxisome proliferator-activated receptor gamma knockout causes insulin resistance in fat and liver but not in muscle. Proc Natl Acad Sci U S A 100: 15712–15717. 14660788 10.1073/pnas.2536828100 PMC307633

[34] Estall JL, Kahn M, Cooper MP, Fisher FM, Wu MK, et al (2009) Sensitivity of lipid metabolism and insulin signaling to genetic alterations in hepatic peroxisome proliferator-activated receptor-gamma coactivator-1alpha expression. Diabetes 58: 1499–1508. 19366863 10.2337/db08-1571 PMC2699879

[35] Burgess SC, Leone TC, Wende AR, Croce MA, Chen Z, et al (2006) Diminished hepatic gluconeogenesis via defects in tricarboxylic acid cycle flux in peroxisome proliferator-activated receptor gamma coactivator-1alpha (PGC-1alpha)-deficient mice. J Biol Chem 281: 19000–19008. 16670093 10.1074/jbc.M600050200 PMC3047410

[36] Nagai Y, Yonemitsu S, Erion DM, Iwasaki T, Stark R, et al (2009) The role of peroxisome proliferator-activated receptor gamma coactivator-1 beta in the pathogenesis of fructose-induced insulin resistance. Cell Metab 9: 252–264. 19254570 10.1016/j.cmet.2009.01.011 PMC3131094

