# Supplementary figures and images for: Liver-Specific PGC-1beta Deficiency Leads to Impaired Mitochondrial Function and Lipogenic Response to Fasting-Refeeding
Source: PLoS One. 2012 Dec 28;7(12):e52645. doi: 10.1371/journal.pone.0052645 (PMC3532159; doi:10.1371/journal.pone.0052645)

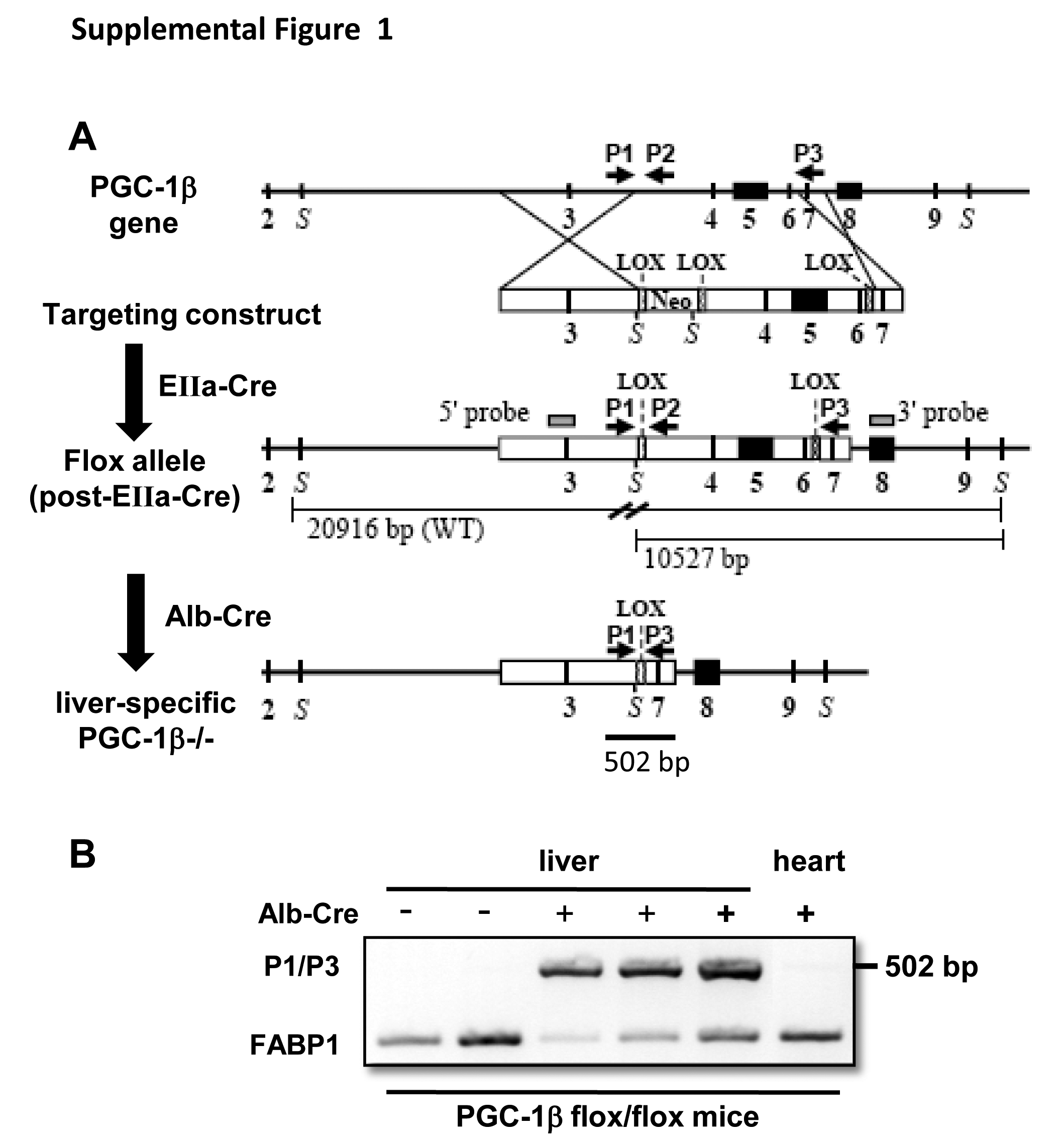

Supplement: Figure S1 — Liver-specific deletion of PGC-1β. [A] The targeting construct and strategy for conditional ablation of PGC-1β is schematized. [B] The gel at right shows the results of PCR analyses using the P1 and P3 primers in the schematic at left and DNA isolated from liver of 6 week old PGC-1β fl/fl mice that were expressing Cre recombinase under the control of the liver specific albumin promoter or were non-transgenic for Cre recombinase. Cardiac DNA from one of the transgenic mice is shown as a control. (TIFF) [file pone.0052645.s001.tiff]
